# Comprehensive Diagnosis of Abnormal Vaginal Discharge Using qPCR-Based Microbial Dysbiosis Indices

**DOI:** 10.3390/diagnostics16132075

**Published:** 2026-07-02

**Authors:** Petra Vovko, Vesna Fabjan Vodušek, Matjaž Retelj, Barbara Sodec, Martina Bučar, Jasna Kostanjšek, Marijana Klarič Kamin, Veronika Testen, Nataša Tul Mandić

**Affiliations:** 1 Department of Medical Microbiology, National Laboratory of Health, Environment and Food, Mej vrti 5, 8000 Novo Mesto, Slovenia; barbara.sodec@nlzoh.si; 2 Department of Perinatology, Division of Gynecology, University Medical Center Ljubljana, Šlajmerjeva 3, 1000 Ljubljana, Slovenia; vesna.fabjan@mf.uni-lj.si (V.F.V.); natasa.tul@guest.arnes.si (N.T.M.); 3 Department for Microbiological Analysis of Food, Water, and Other Environmental Samples, National Laboratory of Health, Environment and Food, Mej vrti 5, 8000 Novo Mesto, Slovenia; matjaz.retelj@nlzoh.si; 4 Community Health Center Novo Mesto, Kandijska Cesta 4, 8000 Novo Mesto, Slovenia; martina.bucar@zd-nm.si (M.B.);

**Keywords:** vaginal discharge, bacterial vaginosis, aerobic vaginitis, candida, sexually transmitted infections

## Abstract

**Background/Objectives**: Abnormal vaginal discharge (AVD) is a common complaint among women of reproductive age, often involving multiple, overlapping etiologies, most commonly bacterial vaginosis (BV), vulvovaginal candidiasis (VVC), aerobic vaginitis (AV), and sexually transmitted infections (STIs). We aimed to evaluate a syndromic diagnostic approach by developing qPCR-derived dysbiosis indices for BV, VVC, and AV, subsequently comparing their performance against established reference methods and clinician-assigned diagnoses. **Methods**: Vaginal swabs were collected in a case–control design from 74 symptomatic and 64 asymptomatic women at two clinics in Slovenia. Commercial qPCR assays quantified the microbial species associated with AVD. Relative abundances were integrated into novel dysbiosis indices. Diagnostic performance was validated against the Nugent scoring system (for BV), semiquantitative Candida culture with clinical symptoms (for VVC), and Hay–Ison criteria (for AV). **Results**: In this internally validated study, dysbiosis indices demonstrated high agreement with their respective reference tests and outperformed clinician-assigned diagnoses across all three conditions. The syndromic approach further revealed that mixed etiologies were frequent, leading to a diagnostic resolution for this patient subset. **Conclusions**: qPCR-based microbial dysbiosis indices offer a robust alternative to microscopy, particularly in settings where microscopy is not routinely performed. This method improves the accuracy of AVD evaluation and supports more targeted clinical management.

## 1. Introduction

Abnormal vaginal discharge (AVD) is a common complaint among women of reproductive age, and an accurate diagnosis is crucial for effective treatment and prevention of recurrence. The most common underlying causes include bacterial vaginosis (BV; 22% to 50% of cases); vulvovaginal candidiasis (VVC; 17–39% of cases); aerobic vaginitis (AV; 7–12% of cases); and sexually transmitted infections (STIs), especially trichomoniasis (4–35% of cases), either alone or in combination. However, in 24–40% of women with AVD, the etiology remains unidentified, and misdiagnosis has been proposed as a major reason why around 40% of patients revisit the clinic with persistent symptoms after treatment [1,2,3].

International guidelines, including the European (International Union against Sexually Transmitted Infections/World Health Organization) Guideline on the Management of Vaginal Discharge and the CDC Sexually Transmitted Infections Treatment Guidelines, emphasize microscopy-based methods as key diagnostic tools in AVD [3,4]. Microscopy is considered the gold standard for diagnosing BV and AV. However, implementing guideline-recommended microscopy into general practice in Slovenia has proven to be quite challenging [5,6]. Therefore, an alternative standardized diagnostic modality that can address all potential AVD causes, particularly BV, VVC, and AV, could have a significant influence on clinical management.

It has been shown that qPCR methods can lead to more effective management strategies in BV [7,8,9]. Some qPCR methods rely on the detection or quantification of bacteria in vaginal secretions and determine positivity based on fixed cut-off values. More recent strategies acknowledge that BV, and possibly AV, result from a shift from a healthy, *Lactobacillus*-dominated vaginal microbiota to a dysbiotic microbial community. Accordingly, these approaches use qPCR-derived relative abundances of specific taxa to calculate microbial dysbiosis indices, similar to the diagnostic methods used in other conditions characterized by polymicrobial dysbiosis [10,11]. qPCR-based dysbiosis indices for BV and AV have been successfully validated in previous studies [12,13,14,15,16,17,18].

Despite this progress, the value of qPCR-based diagnostics in simultaneous assessment of the most common causes of AVD—BV, VVC, AV, and STI—in general clinical practice compared to the current standard of care remains unclear.

This study aimed to develop and evaluate a comprehensive laboratory workflow for women presenting with AVD. We developed novel dysbiosis indices for BV, VVC, and AV, validating their performance against established reference methods. Furthermore, we assessed the diagnostic value of integrating STI screening and compared the accuracy of these indices with clinician diagnoses formulated based on examinations and in-clinic tests to determine their potential for enhancing routine care.

## 2. Materials and Methods

### 2.1. Study Design and Sample Population

We conducted a case–control study in two general population clinics in Slovenia between October 2015 and February 2016. Consecutive women presenting to their primary care gynecologist with genital complaints were invited to participate and formed the AVD group. The subjects were not reimbursed for participating in the study. Individuals were eligible for the AVD group if they reported one or more of the following symptoms: AVD (as subjectively assessed by the patient); unpleasant odor; burning sensation; itching; and dyspareunia. Simultaneously, a control group was recruited that consisted of women attending the same clinics for routine gynecological exams during the study period who did not report genital complaints. The inclusion criteria for both groups were as follows: non-pregnant women with regular menstrual cycles; age 18–45 years; no use of systemic or local antibiotics in the preceding 4 weeks; and no use of intravaginal products in the preceding 24 h.

Vaginal swabs were obtained using an unmoistened speculum following a visual examination of external genitalia and before any other vaginal procedure. Two vaginal swabs were then taken from the upper third of the lateral vaginal wall and the posterior fornix using a polyurethane swab (MWE, Corsham, UK) and a flocked nylon swab (Copan, Brescia, Italy). The polyurethane swab was placed in 1 mL of Amies transport medium for microscopic examination and cultivation, while the nylon swab was placed in 1 mL of transport medium for molecular methods. The swabs were refrigerated for a maximum of 24 h before laboratory procedures were conducted. After swabbing, clinical tests were performed.

### 2.2. Clinical Testing and Diagnosis

In-clinic tests comprised assessing the vaginal discharge, measuring the vaginal pH, and performing the amine odor test. Vaginal pH was measured by transferring vaginal secretions (avoiding cervical mucus) to pH indicator strips (pH 3.6–6.1; Macherey-Nagel, Düren, Germany). An amine (whiff) test was performed by adding 1–2 drops of 10% KOH to a portion of vaginal fluid on a glass slide and assessing for a fishy, amine-like odor. In-clinic microscopy-based diagnostic methods (wet mount) for a comprehensive assessment according to Amsel criteria are not practiced in Slovenia.

Gynecologists established a clinical diagnosis based on patient history, reported symptoms, and clinical examination findings, without knowledge of the laboratory test results.

### 2.3. Laboratory Tests

Vaginal swabs were tested for BV, VVC, and AV using qPCR assays and reference methods, and for STIs using a multiplex qPCR panel.

AmpliSens qPCR assays (Central Research Institute for Epidemiology, Moscow, Russia) were used to measure the abundances (log_10_ copies/mL swab) of bacteria associated with BV, VVC, and AV. The assays had been clinically or technically validated [13,14,19,20] and underwent verification in our laboratory prior to use.

Microbial genomic DNA was extracted from 250 µL of the vortexed nylon swabs, supplemented with 25 µL of the amplification control, using the Arrow automatic extraction system and the Viral NA reagent (DiaSorin, Saluggia, Italy). All qPCR reactions were performed using a Bio-Rad CFX96 IVD system (Bio-Rad Laboratories, Hercules, CA, USA). The Cq threshold values and abundances were determined according to the assay manufacturer’s instructions.

For BV, the AmpliSens Florocenosis/Bacterial Vaginosis-FRT CE-IVD assay quantified *Gardnerella vaginalis*, *Fannyhessea vaginae* (formerly *Atopobium vaginae*), *Lactobacillus* spp., and total bacteria (*Bacteria* domain). Their abundances, expressed as log_10_ copies/mL swab, are denoted in the subsequent text as g, a, l, and t, respectively.

For VVC, the AmpliSens Florocenosis Candida-FRT assay quantified *Candida albicans*, *Nakaseomyces glabrata* (formerly *Candida glabrata*), *Pichia kudriavzevii* (formerly *Candida krusei*), *Candida parapsilosis*, and *Candida tropicalis*. The abundance of the *Candida* species, denoted as c, was calculated as the log_10_-transformed sum of all five *Candida* species levels per mL swab.

For AV, the AmpliSens Florocenosis Aerobes-FRT assay quantified *Enterobacteriaceae*, *Staphylococcus* spp., and *Streptococcus* spp. The abundances of the three microbial groups were summed, log_10_-transformed, and denoted as ess.

Reference tests for BV and VVC were chosen in accordance with the European (IUSTI/WHO) Guideline on the Management of Vaginal Discharge [3]. These include Gram-stained microscopy with Nugent scoring (NS) for BV and medium or heavy growth of Candida spp. in culture, combined with symptoms for VVC. The reference test used for AV was Gram-stained microscopy with Hay–Ison criteria [21].

Gram-stained slides for NS and Hay–Ison scoring were prepared by staining 50 µL of the polyurethane swab fluid using the Previ system (bioMerieux, Marcy-l’Étoile, France). The slides were categorized into three groups—normal (score 0–3), intermediate (score 4–6), and bacterial vaginosis (score 7–10)—according to Nugent et al. [22]. Samples that met the Hay–Ison Grade 4 criteria were classified as positive for AV.

Candida culture was performed by inoculating 50 µL of polyurethane swab liquid onto Candida Chrom agar (bioMerieux), followed by incubation at 36 °C in ambient air for 48 h. Colonies were then identified according to the manufacturers’ instructions and counted. Growth was categorized as light (1–29 colonies), medium (30–299), or heavy (≥300).

STI testing was performed using the Anyplex II STI-7 assay (Seegene, Seoul, Republic of Korea) on 5 µL of DNA extract to detect *Chlamydia trachomatis*, *Neisseria gonorrhoeae*, *Mycoplasma genitalium*, *Mycoplasma hominis*, and *Trichomonas vaginalis.* Positivity was interpreted automatically using the Seegene Viewer software (version 3). This assay has been subjected to prior clinical validation [23,24].

### 2.4. Development of the Dysbiosis Indices

qPCR targets were combined into candidate dysbiosis indices, i.e., single numerical values that allow for the classification of patients as positive or negative for a given condition based on a cut-off value. In parallel, the diagnostic performance of individual relative microbial abundances (levels expressed in log_10_ per mL swab) was also assessed.

Several candidate indices were explored during the data analysis phase. Because microbial abundances are expressed as log_10_ copies/mL swab, the subtraction of two abundances corresponds to the logarithm of their ratio. For example, the BV index g-l was calculated as the abundance of *G. vaginalis* (g) minus the abundance of *Lactobacillus* spp. (l), equivalent to log_10_(*G. vaginalis*/*Lactobacillus* spp.). Similarly, (ga)-l was calculated as the combined abundance of *G. vaginalis* and *F. vaginae* (ga) minus the abundance of *Lactobacillus* spp. (l), equivalent to log_10_[(*G. vaginalis* + *F. vaginae*)/*Lactobacillus* spp.].

The potential candidate dysbiosis indices and relative abundance metrics were validated using training datasets defined by reference test results. For BV, the training dataset was formed regardless of the enrollment group, as asymptomatic BV was anticipated both in the healthy control (HC) group and the AVD group. The BV-positive training dataset included all participants (from both the AVD and HC groups) with an NS ≥ 7. The BV-negative training dataset comprised all participants (from the AVD and HC groups) with NS ≤ 3. For VVC, the VVC-positive training dataset included subjects from the AVD group with a clinical diagnosis of VVC and medium or heavy growth of *Candida* in culture. The VVC-negative set consisted of all healthy controls, irrespective of culture results, acknowledging that *Candida* colonization can occur in asymptomatic women. For AV, the AV-positive and AV-negative training datasets consisted of participants with Hay–Ison scores of 4 and 1, respectively, again irrespective of study group.

Receiver operating characteristic (ROC) analysis was used to determine the cut-off values and corresponding sensitivities and specificities of each candidate dysbiosis index and relative microbial abundance measure. In the ROC analysis, the criterion for selecting optimal cut-off values for the BV and VVC dysbiosis indices, which represent more prevalent conditions, was achieving sensitivity >94% and specificity >90%. In contrast, because AV is a low-prevalence condition, high specificity was prioritized to minimize false-positive results. Therefore, a specificity of at least 96% was required when selecting the optimal AV cut-off.

### 2.5. Validation of the Dysbiosis Indices

To evaluate the diagnostic performance of the dysbiosis indices, an internal validation within the study population was performed on the complete AVD cohort. The cut-offs established on the training subset were applied to this complete subject group, and the sensitivity, specificity, positive predictive value (PPV), and negative predictive value (NPV) were calculated against the reference tests. Because the cut-offs were derived and assessed within the same overall study population, this procedure should be interpreted as internally validated, rather than an independent external validation.

### 2.6. Statistical Analysis

Group comparisons and ROC analyses were performed in GraphPad Prism 10.2 (GraphPad Software, San Diego, CA, USA). Fisher’s exact test or unpaired *t*-tests were applied as appropriate. The Wilson–Brown method was used to calculate confidence intervals for diagnostic accuracy metrics, and significance was set at *p* < 0.05.

## 3. Results

### 3.1. Study Population

The study enrolled a total of 138 participants: 74 in the AVD group and 64 in the HC group. Demographic and clinical characteristics, including age, underlying diseases, allergies, long-term medication use, and type of contraception, did not differ significantly between groups (Table 1).

**Figure 1:**
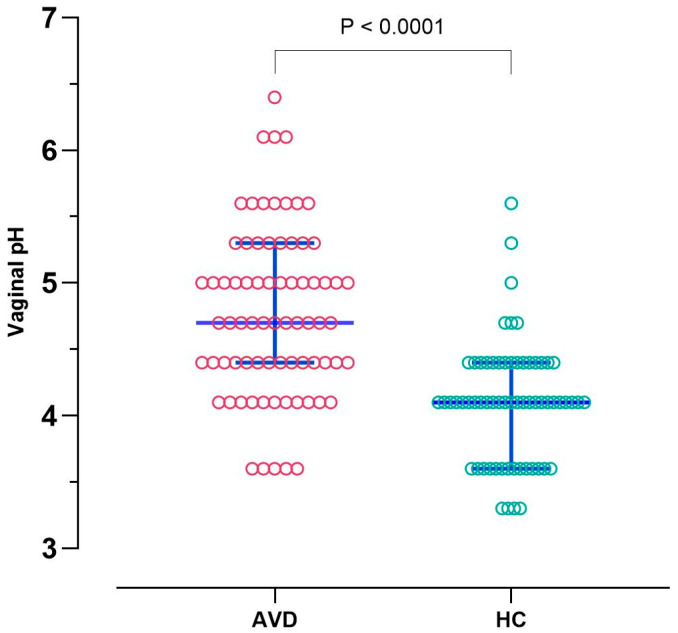
Distribution of vaginal pH values in altered vaginal discharge (AVD, red circles) and healthy control (HC, green circles) subjects, measured at the clinical examination (each circle represents one subject). The blue handles represent the median and the interquartile range. The groups showed a statistically significant difference (unpaired *t*-test).

The symptoms most frequently reported by women in the AVD group, leading to a visit to the gynecologist, were changes in vaginal discharge, either isolated or accompanied by an unpleasant smell, itching, or painful sensations. Clinical findings and the results of in-clinic tests in the AVD and HC groups are reported in Table S1. Vaginal pH was significantly higher in the AVD group (median 4.7, interquartile range [IQR] 4.4–5.2) than in the HC group (median 4.1, IQR 3.6–4.4; see Figure 1).

### 3.2. Reference Test Results and STI Panel Detections

The results for reference laboratory tests and multiplex STI panel detections are shown in Table 2. A total of 31.1% of AVD patients and 10.9% of HC subjects, respectively, had an NS of 7–10. As the healthy controls did not report genital complaints, these findings represent asymptomatic BV, as measured by the reference method. Intermediate microbiota (NS 4–6) was more frequent in the AVD group. Hay–Ison grading was available for 45 AVD patients and 46 controls. AV was detected in three AVD patients (4.1%) and two HC subjects (3.1%), and medium or heavy *Candida* growth was present in 39.2% of AVD patients and 17.2% of HC subjects, respectively. In the multiplex STI panel, detected species included *Chlamydia trachomatis*, *Trichomonas vaginalis*, and *Mycoplasma hominis*.

### 3.3. qPCR Abundances of Targeted Taxa

**Figure 2:**
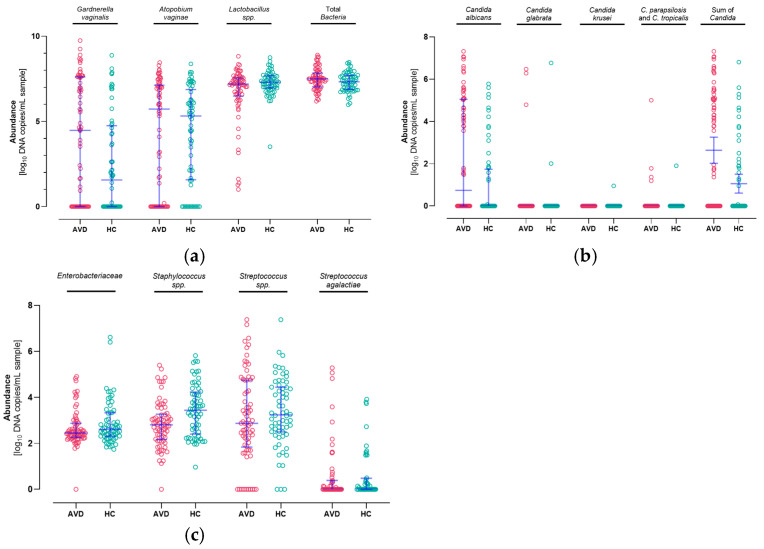
Scatter plots of the abundances of each microbial group targeted in the qPCR panels for BV (**a**), VVC (**b**), and AV (**c**) in altered vaginal discharge (AVD) and healthy control (HC) subjects. The blue horizontal lines represent the median and the interquartile range.

Figure 2 shows the distributions of the qPCR-derived abundances of taxa associated with BV, VVC, and AV in the AVD and HC groups. For many targets, there was extensive overlap between groups, supporting the need for composite dysbiosis indices rather than simple presence/absence or single cut-offs. Therefore, various combinations of qPCR-derived abundances of targeted microbial taxa were evaluated to find the dysbiosis indices for BV, VVC, and AV with the highest discriminatory power.

### 3.4. BV qPCR Dysbiosis Index Development

**Figure 3:**
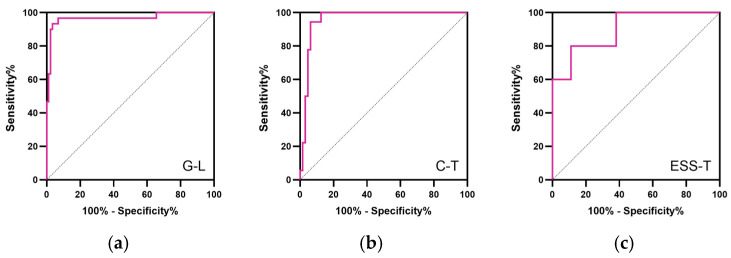
Receiver operating characteristic curves of the best-performing dysbiosis indices in the training datasets for BV (**a**), VVC (**b**), and AV (**c**).

For the development of the BV dysbiosis index, a total of 30 subjects from the AVD and HC groups with NS ≥ 7 were included in the BV-positive training dataset, and a total of 87 subjects from both groups with NS ≤ 3 were included in the BV-negative training dataset (Supplementary Figures S1 and S2). We evaluated several candidate dysbiosis indices for BV prediction by transforming qPCR-derived abundances into log-ratio indices: g-l, a-l, (ga)-l, and l-t. Their diagnostic performances are reported in Supplementary Table S2. Among these, the g-l index demonstrated the best performance. Using the selected cut-off value of >−1.339, the g-l index achieved 96.7% sensitivity and 93.1% specificity for BV diagnosis compared with the reference NS (Table 3 and Figure 3).

### 3.5. VVC qPCR Dysbiosis Index Development

For the development of the VVC dysbiosis index, all patients with a clinical diagnosis of VVC and medium or heavy growth of *Candida* in culture (*N* = 18) were included in the VVC-positive training dataset, and all 64 HC subjects were included in the VVC-negative training dataset (Supplementary Figure S3 and Table S3).

The dysbiosis index that showed the best discriminatory power was the relative abundance of tested *Candida* species (c) to the total bacteria, i.e., the c-t ratio. The optimal cut-off value for the c-t ratio was −3.065, with 94.4% sensitivity and 93.8% specificity (Table 3 and Figure 3).

### 3.6. AV qPCR Dysbiosis Index Development

For the development of the AV dysbiosis index, the 3 patients and 2 controls with Hay–Ison scores of 4 were included in the AV-positive training dataset, and the 26 patients and 37 controls with Hay–Ison scores of 1 were included in the AV-negative dataset (Supplementary Figure S4; Supplementary Table S4). The optimal dysbiosis index selected for AV was the relative abundance of ess to the total bacteria, i.e., the ess-t ratio. The cut-off value was adjusted to the low prevalence of AV due to the risk of generating numerous false-positive results. Therefore, a more conservative cut-off of −0.889 was selected as optimal, providing 60% sensitivity and 98.4% specificity (Table 3).

### 3.7. Validation of the qPCR Dysbiosis Indices

The diagnostic performance of the developed qPCR-based microbial indices was internally evaluated within the AVD group through comparison with reference laboratory tests (Nugent score for BV; clinical presentation and medium-to-heavy growth of Candida for VVC; and Hay–Ison criteria for AV). Performance metrics were also calculated for the clinical diagnoses formulated based on clinical examination and in-clinic tests. As detailed in Table 4, the molecular dysbiosis indices demonstrated promising diagnostic accuracy in this internally validated study population for BV and VVC. Notably, the indices significantly outperformed clinical diagnoses, particularly in the detection of a combination of BV + VVC. For AV, all three reference-positive AVD cases were identified via the ess-t index; however, this estimate should be interpreted as exploratory because it was based on only three AV-positive cases in the AVD group.

### 3.8. Combinations of Causes of Altered Vaginal Discharge

Various combinations of conditions were observed in the AVD group, with the most common being BV + VVC (6.7%) and BV + multiplex STI panel detections (12.2%). The qPCR-based dysbiosis indices correctly identified four of the five cases of BV + VVC, while one was classified as VVC-only (Table 5). In contrast, clinical diagnosis failed to identify mixed etiologies within this group.

STI-associated organisms were detected in 15 AVD patients. Three multiplex STI panel detections occurred in patients with negative reference tests, indicating that they were the primary cause of symptoms. Among those with reference-confirmed BV (*N* = 18), co-detections included *Mycoplasma hominis* (*N* = 6), *Chlamydia trachomatis* (*N* = 3), and *Trichomonas vaginalis* (*N* = 2).

## 4. Discussion

In this study, we developed and evaluated microbial dysbiosis indices for the diagnosis of BV, VVC, and AV and implemented them within a syndromic molecular diagnostic workflow for women presenting with AVD. The dysbiosis indices demonstrated excellent diagnostic performance when compared with established reference laboratory methods. Importantly, the molecular indices substantially outperformed clinician-based diagnosis, supporting their use for differential diagnosis of vaginal disorders.

These findings reinforce the concept that BV and other vaginal disorders are best understood as ecological disturbances in the vaginal microbiota. Earlier molecular approaches focused on the detection or quantification of individual microorganisms, but research has increasingly demonstrated that diagnostic accuracy improves when microbial measurements are interpreted in relation to the overall community structure, especially the relative abundance of *Lactobacillus* species compared with dysbiosis-associated taxa.

Our approach extends the existing literature by combining quantitative measurements of microbial targets with dysbiosis indices for three major causes of vaginitis and integrating them into a unified diagnostic workflow. The diagnostic performance of the BV dysbiosis index in this study, g-l, reflecting the ratio of *G. vaginalis* to *Lactobacillus* spp., is consistent with the results from other molecular studies. Deng et al. demonstrated that the ratio between *Lactobacillus crispatus* and *G. vaginalis* provides a highly sensitive and specific indicator of BV [16]. Plummer et al. similarly demonstrated that the ratio between *G. vaginalis*, *A. vaginae*, and the total bacterial load is very sensitive [15]. Rumyantseva used a BV dysbiosis index adjusted to the abundance of lactobacilli but reported lower diagnostic accuracy compared to the present study [14].

In our cohort, 10.9% of subjects in the HC group had Nugent scores consistent with BV despite the absence of genital complaints. This was anticipated during the study design; therefore, all Nugent-positive and Nugent-negative participants, irrespective of enrollment group, were included in the development of the BV dysbiosis index. We considered this appropriate because the aim of the BV index was to capture BV-associated microbial dysbiosis, defined by the reference method rather than symptom status alone. At the same time, this means that the BV dysbiosis index should be interpreted primarily as a marker of microbiological BV, while the clinical relevance of BV detected in asymptomatic women should be interpreted in the appropriate clinical context.

Diagnosis of VVC remains challenging because *Candida* species frequently colonize the vagina without causing symptomatic infection. While fungal culture remains the traditional reference standard, molecular assays are increasingly used due to their ability to accelerate diagnosis and improve sensitivity. However, one limitation of PCR-based methods is the difficulty of defining quantitative thresholds that differentiate asymptomatic colonization from clinically significant infection [2]. In our study, the VVC dysbiosis index, based on the ratio of *Candida* abundance relative to total bacterial load, demonstrated high sensitivity while maintaining good specificity. These results suggest that incorporating bacterial community context improves diagnostic discrimination compared with direct quantification of *Candida* alone.

The performance of the AV dysbiosis index was more limited and should be interpreted with particular caution, reflecting both the small number of AV-positive cases in the cohort and the heterogeneity of this condition. Although the ess-t index identified all three reference-positive AV cases in the AVD group, this resulted in an apparently perfect sensitivity estimate with a wide confidence interval and should not be considered a definitive estimate of diagnostic performance. Nevertheless, our findings align with previous work by Rumyantseva et al., who proposed a PCR-based AV index incorporating aerobic bacteria such as *Enterobacteriaceae*, *Staphylococcus*, and *Streptococcus*, normalized to lactobacilli abundance [13].

An important observation in this study was the frequent occurrence of overlapping etiologies. Several patients presented with combinations of BV, VVC, and STI-associated organisms, highlighting the complexity of vaginal dysbiosis. This limits the utility of targeted diagnostics based solely on clinical suspicion, and multiplex molecular panels that simultaneously evaluate multiple pathogens and microbial markers may represent a practical solution for routine clinical diagnostics. However, the clinical interpretation of individual organisms requires caution. In particular, *Mycoplasma hominis* was included because it was a target of the multiplex STI panel used, but it should not be interpreted as a classical STI pathogen [25]. In our cohort, *M. hominis* was detected mainly as a co-detection, especially in participants with BV, suggesting that it may reflect dysbiosis-associated colonization or opportunistic overgrowth rather than an independent cause of symptoms.

Furthermore, qPCR-based syndromic diagnostics offer several advantages over conventional microscopy. Although molecular testing requires access to laboratory infrastructure, it eliminates the need for specialized microscopy expertise and reduces subjectivity in interpretation. This may be particularly beneficial in primary care settings where microscopy is not routinely available.

This study has several limitations. First, the number of AV-positive cases was very small: AV was identified in only five participants in the total study population, and in only three symptomatic women in the AVD validation cohort. Therefore, the diagnostic performance estimates for AV should be interpreted with caution, because even apparently excellent performance is based on very few positive cases and is associated with limited precision, as reflected by the wide confidence intervals. Accordingly, the AV findings should be considered preliminary and require confirmation in larger studies.

Secondly, the dysbiosis indices were developed and internally validated within the same overall study population, and no independent external validation cohort was available. Therefore, the reported diagnostic performance may be optimistic, and the proposed cut-off values and indices should be confirmed in an external cohort before broader clinical generalization.

Third, the findings should be interpreted primarily within a defined Slovenian clinical setting and may not be directly generalizable to other geographic, ethnic, behavioral, reproductive, or hormonal populations. Because vaginal microbiome composition is influenced by host and population context, the proposed dysbiosis indices should be externally validated in larger and more diverse cohorts before broader clinical generalization. Despite these limitations, our findings suggest that qPCR-derived dysbiosis indices, when part of a syndromic panel that also includes STI testing, can improve the diagnostic evaluation of women with AVD.

We used qPCR in this study, which is increasingly being replaced by digital PCR in the microbial ecology field. Digital PCR offers highly precise absolute quantification, robust multiplexing, and potentially lower labor costs. Future research should explore the application of digital PCR to dysbiosis indices for AVD, including possible improvements in accuracy, reproducibility, and cost-effectiveness.

## 5. Conclusions

The results of this study demonstrate that qPCR-derived microbial dysbiosis indices can provide accurate and objective diagnoses of BV, VVC, and AV within the clinical context studied. By integrating these indices into a syndromic molecular testing framework, clinicians may achieve a more comprehensive and reliable evaluation of women presenting with AVD. Such approaches align with recent advances in microbiome-based diagnostics and have the potential to improve both the diagnostic accuracy and clinical management of AVD.

## Figures and Tables

**Table 1:** Comparison of the patients and the controls by age and medical history data.

		Altered Vaginal Discharge *N* = 74	Healthy Controls *N* = 64	*p*-Value for the Difference
Age, median (interquartile range)		30.0 (23.8–35.0)	32.5 (27.0–37.8)	0.11
Medical history data, *N* (%)				
Underlying diseases	Yes	6 (8.1)	6 (9.4)	>0.99
	No	68 (91.9)	58 (90.6)	
Allergies	Yes	13 (17.3)	8 (12.5)	0.48
	No	62 (82.7)	56 (87.5)	
Long-term medication use	Yes	6 (8.1)	6 (9.4)	0.79
	No	68 (91.9)	58 (90.6)	
Type of contraception	None	46 (62.1)	39 (60.9)	0.61
	Condom	5 (6.8)	6 (9.4)	
	Intrauterine device	5 (6.8)	7 (12.5)	
	Oral hormonal contraception	18 (24.3)	11 (17.2)	

For age, the median and interquartile range are provided. For binary variables, the number and percentage of subjects in the group are given. The groups did not differ significantly in age (unpaired *t*-test) and medical history data (Fisher’s exact test).

**Table 2:** The results of reference laboratory tests and the multiplex STI panel.

	Altered Vaginal Discharge *N* = 74	Healthy Controls *N* = 64
Nugent score for BV, *N* (%)		
0–3 (normal)	36 (48.6)	51 (79.7)
4–6 (intermediate microbiota)	15 (20.3)	6 (9.4)
7–10 (BV)	23 (31.1)	7 (10.9)
Hay–Ison grade for AV, *N* (%)		
0 (use of antibiotics)	0 (0.0)	0 (0.0)
1 (normal state)	26 (35.1)	37 (57.8)
2 (intermediate state)	0 (0.0)	0 (0.0)
3 (BV)	16 (21.6)	7 (10.9)
4 (AV)	3 (4.1)	2 (3.1)
Not completed	29 (39.2)	18 (28.2)
Growth of *Candida* in culture, *N* (%)		
Not detected	41 (55.4)	49 (76.5)
Light	4 (5.4)	4 (6.3)
Medium	13 (17.6)	5 (7.8)
Heavy	16 (21.6)	6 (9.4)
Medium or heavy	29 (39.2)	11 (17.2)
Multiplex STI panel detections, *N* (%)		
*Chlamydia trachomatis*	5 (6.8)	1 (1.6)
*Trichomonas vaginalis*	2 (2.7)	0 (0.0)
*Mycoplasma hominis*	8 (10.8)	4 (6.3)
*Mycoplasma genitalium*	0 (0.0)	0 (0.0)
*Neisseria gonorrhoeae*	0 (0.0)	0 (0.0)
None of the above	59 (79.7)	59 (92.1)

**Table 3:** Diagnostic performance of the best-performing dysbiosis indices for BV, VVC, and AV in the training datasets.

Condition	Dysbiosis Index	Cut-Off Value	Sensitivity (%)	Specificity (%)	AUC (95% CI)
BV	g-l	>−1.339	96.7	93.1	0.9667 (0.9224–1.000)
VVC	c-t	>−3.065	94.4	93.8	0.9583 (0.9161–1.000)
AV	ess-t	>−0.855	60.0	98.4	0.9016 (0.7656–1.000)

**Table 4:** Comparison of diagnostic performance between the developed qPCR-based microbial dysbiosis indices and clinical diagnosis in patients with altered vaginal discharge.

Condition	Method	Sensitivity (95% CI)	Specificity (95% CI)	PPV (%)	NPV (%)
BV	Dysbiosis index g-l	95.7 (79–99.8)	91.7 (78.2–97.1)	88.0	97.1
	Clinical diagnosis	47.8 (29.2–67.0)	100.0 (90.4–100.0)	100.0	75.0
VVC	Dysbiosis index c-t	94.4 (74.2–99.7)	80.4 (68.2–88.7)	60.7	97.8
	Clinical diagnosis	100.0 (82.4–100.0)	80.4 (68.2–88.7)	62.1	100.0
AV	Dysbiosis index ess-t	3/3; 100.0 (43.9–100.0)	42/42; 100.0 (91.6–100.0)	100.0	100
	Clinical diagnosis	0.0 (0.0–56.2)	92.9 (81–97.5)	0.0	92.9
BV + VVC	Concurrent positivity of g-l and c-t	80.0 (37.6–99)	91.3 (82.3–96.0)	40.0	98.4
	Clinical diagnosis	0.0 (0.0–43.5)	98.6 (92.2–99.9)	0.0	93.2

Reference standard tests: Nugent score for BV; clinical presentation of VVC and medium or heavy growth of Candida for VVC; and Hay–Ison criteria for AV.

**Table 5:** Concordance between reference tests, qPCR-based microbial dysbiosis indices, and clinical diagnoses in the AVD group, including STI detections. Data are presented as the number of patients.

Reference Test Result		BV-Only *N* = 18	BV + VVC *N* = 5	VVC-Only *N* = 13	AV *N* = 3	Negative *N* = 35
Multiplex STI panel Detections *	*Chlamydia* *trachomatis*	3				2
	*Trichomonas vaginalis*	2				
	*Mycoplasma hominis*	6		1		1
Dysbiosis index results	BV only	16				2
	BV + VVC	2	4	3		1
	VVC-only		1	9		7
	AV				3	1
	Negative			1		24
Clinical diagnosis	BV-only	10				1
	BV + VVC	1				
	VVC-only	1	5	13		9
	AV	3				5
	Inconclusive	3			3	20

* Some patients had multiple STI panel detections. Shaded cells indicate concordant classifications between the reference test result shown in the column header and the corresponding dysbiosis index result or clinical diagnosis.

## Data Availability

The data presented in this study are available on request from the corresponding author due to privacy considerations.

## Supplementary Materials

The following supporting information can be downloaded at https://www.mdpi.com/article/10.3390/diagnostics16132075/s1, Table S1: Detailed clinical characteristics and in-clinic test results of study participants. Figure S1: Scatter plots of the abundances of BV-associated microbial groups in different Nugent classes. The blue horizontal lines represent the median and the interquartile range. Figure S2: Scatter plots and receiver operating characteristic curves of different candidate dysbiosis indices for BV in the BV training dataset. The red dotted lines in the scatter plots represent the selected cut-off values. The blue horizontal lines represent the median and the interquartile range. Table S2: Diagnostic performance of different candidate dysbiosis indices for BV in the BV training dataset. Figure S3: Scatter plots and receiver operating characteristic (ROC) curves for *Candida* abundance and two candidate VVC dysbiosis indices in the VVC training dataset. The red dotted lines represent the selected cut-off values. The blue horizontal lines represent the median and the interquartile range. Table S3: Diagnostic performance of *Candida* abundance and two candidate VVC dysbiosis indices in the VVC training dataset. Figure S4: Scatter plots and receiver operating characteristic (ROC) curves for sum of *Enterobacteria*, *Staphylococcus*, and *Streptococcus* (ess) abundance and two candidate AV dysbiosis indices in the AV training dataset. The red dotted lines represent the selected cut-off values. The blue horizontal lines represent the median and the interquartile range. Table S4: Diagnostic performance of sum of *Enterobacteria*, *Staphylococcus*, and *Streptococcus* (ess) abundance and two candidate AV dysbiosis indices in the AV training dataset. Two cut-off values are presented for ess-t, as elaborated in the text.

## Abbreviations

The following abbreviations are used in this manuscript:

qPCR	Quantitative real-time polymerase chain reaction
AVD	Abnormal vaginal discharge
BV	Bacterial vaginosis
VVC	Vulvovaginal candidiasis
AV	Aerobic vaginitis
STI	Sexually transmitted infection
NS	Nugent scoring
HC	Healthy control
ROC	Receiver operating characteristic
AUC	Area under the ROC curve
CI	Confidence interval
