# Supplementary material for: Comprehensive Diagnosis of Abnormal Vaginal Discharge Using qPCR-Based Microbial Dysbiosis Indices
Source: Diagnostics (Basel). 2026 Jul 2;16(13):2075. doi: 10.3390/diagnostics16132075 (PMC13360468; doi:10.3390/diagnostics16132075)
Supplement: Supplementary file 1 [file diagnostics-16-02075-s001.zip › diagnostics-4354887-supplementary.pdf]

diagnostics

Supplementary Tables and Figures

Table S1. Detailed clinical characteristics and in-clinic test results of study participants.

		Altered vaginal discharge N=74	Healthy controls N=64
**Symptoms reported as the reason for the visit,** N (%)			
	Altered discharge	22 (29.7)	
	Itching	1 (1.4)	
	Dyspareunia	2 (2.7)	
	Burning/painful sensation	4 (5.4)	
	Altered discharge + odor	11 (14.9)	
	Altered discharge + itching	9 (12.2)	
	Altered discharge + burning/painful sensation	11 (14.9)	
	Altered discharge + other combinations	14 (18.9)	
**Clinical examination findings,** N (%)			
Vulva	Normal	52 (70.3)	64 (100.0)
	Altered	22 (29.7)	0 (0.0)
Vagina	Normal	11 (14.9)	53 (82.8)
	Altered	63 (85.1)	11 (17.2)
Cervix	Normal	62 (83.8)	63 (98.4)
	Altered	12 (16.2)	1 (1.6)
**Vaginal pH,** median (interquartile range)		4.7 (4.4–5.2)	4.1 (3.6–4.4)
**Positive amine odor test**		15 (20.3)	0 (0.0)

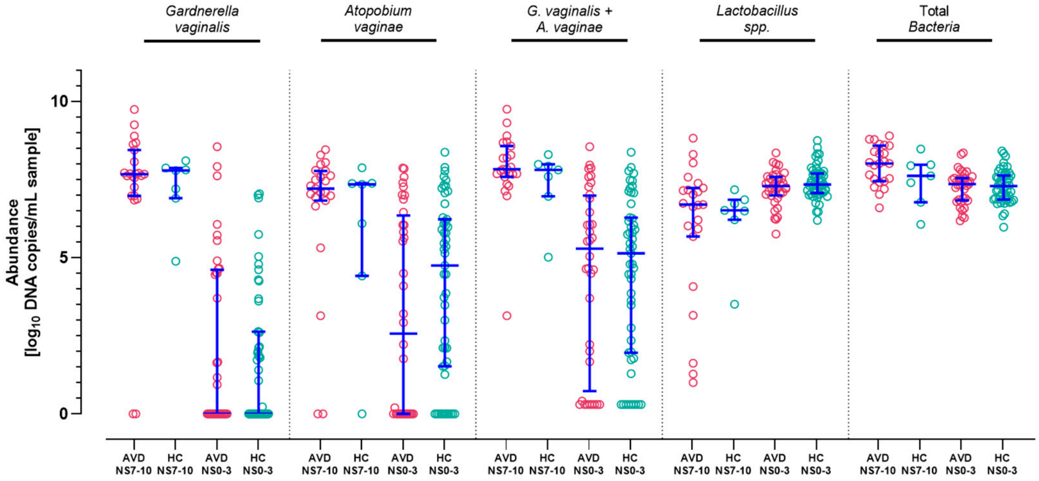

Figure S1. Scatter plots of the abundances of BV-associated microbial groups in different Nugent classes. The blue horizontal lines represent the median and the interquartile range.

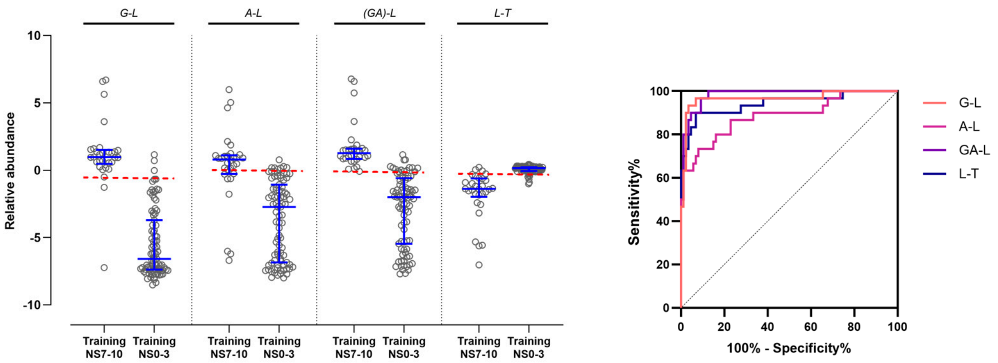

Figure S2. Scatter plots and receiver operating characteristic curves of different candidate dysbiosis indices for BV in the BV training dataset. The red dotted lines in the scatter plots represent the selected cut-off values. The blue horizontal lines represent the median and the interquartile range.

Table S2. Diagnostic performance of different candidate dysbiosis indices for BV in the BV training dataset.

Dysbiosis index	Cut-off	Sensitivity (%)	Specificity (%)	Area under the ROC curve (95% CI)	Likelihood ratio (P value)
G-L	> -1.339	96.7	93.1	0.9667 (0.9224–1.000)	14.0 (<0.0001)
A-L	> -0.0251	73.3	92.0	0.8858 (0.8070–0.9647)	9.1 (<0.0001)
(GA)-L	> 0.1009	93.3	90.8	0.9824 (0.9643–1.000)	7.9 (<0.0001)
L-T	< -0.2635	90.0	93.1	0.9433 (0.8867–0.9998)	13.1 (<0.0001)

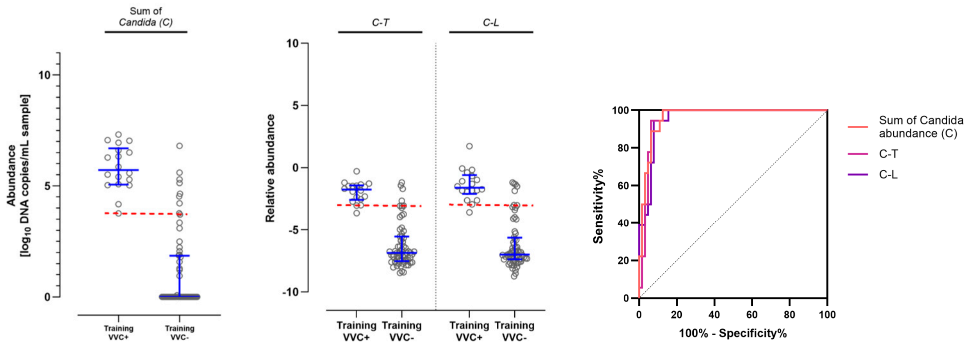

Figure S3. Scatter plots and receiver operating characteristic (ROC) curves for *Candida* abundance and two candidate VVC dysbiosis indices in the VVC training dataset. The red dotted lines represent the selected cut-off values. The blue horizontal lines represent the median and the interquartile range.

Table S3. Diagnostic performance of *Candida* abundance and two candidate VVC dysbiosis indices in the VVC training dataset.

Dysbiosis index	Cut-off	Sensitivity (%)	Specificity (%)	Area under the ROC curve (95% CI)	Likelihood ratio (P value)
Sum of *Candida* (log10 DNA copies/mL sample)	> 3.737	100.0	87.5	0.9644 (0.9288–1.000)	8.0 (<0.0001)
C-T	> -3.065	94.4	93.8	0.9583 (0.9161–1.000)	15.1 (<0.0001)
C-L	> -2.986	94.4	92.2	0.9557 (0.9152–0.9963)	12.1 (<0.0001)

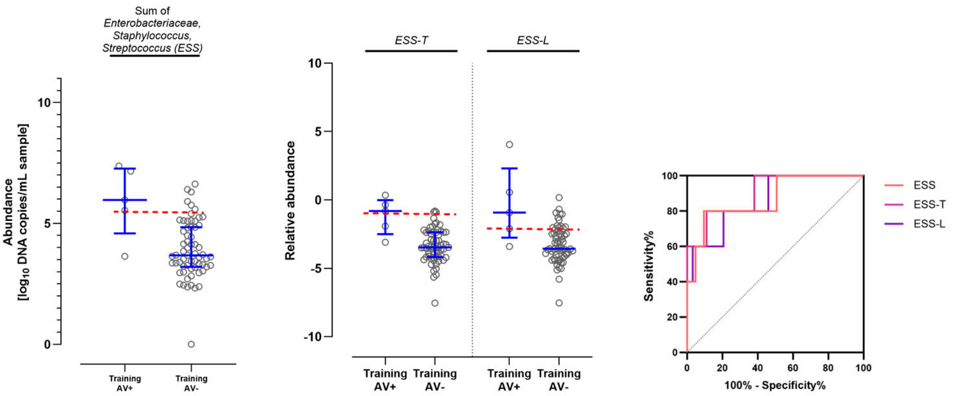

Figure S4. Scatter plots and receiver operating characteristic (ROC) curves for sum of *Enterobacteria, Staphylococcus*, and *Streptococcus* (*ESS*) abundance and two candidate AV dysbiosis indices in the AV training dataset. The red dotted lines represent the selected cut-off values. The blue horizontal lines represent the median and the interquartile range.

Table S4. Diagnostic performance of sum of *Enterobacteria, Staphylococcus*, and *Streptococcus* (*ESS*) abundance and two candidate AV dysbiosis indices in the AV training dataset. Two cut-off values are presented for *ESS-T*, as elaborated in the text.

Dysbiosis index	Cut-off	Sensitivity (%)	Specificity (%)	Area under the ROC curve (95% CI)	Likelihood ratio (P value)
ESS	> 5.471	80.0	90.5	0.8698 (0.6952–1.000)	8.4 (0.0062)
ESS-T	> -1.933	80.0	88.9	0.9016 (0.7656–1.000)	7.2 (0.0030)
ESS-T	> -0.855	60.0	98.4	0.9016 (0.7656–1.000)	37.8 (0.0030)
ESS-L	> -2.136	80.0	79.4	0.8603 (0.6966–1.000)	3.9 (0.0077)
